# Supplementary material for: Effervescent Atomizer as Novel Cell Spray Technology to Decrease the Gas-to-Liquid Ratio
Source: Pharmaceutics. 2022 Nov 9;14(11):2421. doi: 10.3390/pharmaceutics14112421 (PMC9697195; doi:10.3390/pharmaceutics14112421)
Supplement: Supplementary file 1 [file pharmaceutics-14-02421-s001.zip › pharmaceutics-1958634-supplementary.pdf]

Supplementary Data

Effervescent Atomizer as Novel Cell Spray Technology to Decrease the Gas-to-Liquid Ratio

Anja Lena Thiebes [1,2,*,†], Sarah Klein [1,2,†], Jonas Zingsheim [1,3], Georg H. Möller [4], Stefanie Gürzing [4], Manuel A. Reddemann [4], Mehdi Behbahani [3], Stefan Jockenhoevel [1,2,*] and Christian G. Cornelissen [1,5]

1 Department of Biohybrid & Medical Textiles (BioTex), AME—Institute of Applied Medical Engineering, Helmholtz Institute Aachen, RWTH Aachen University, Forckenbeckstraße 55, 52074 Aachen, Germany
2 Aachen-Maastricht Institute for Biobased Materials, Faculty of Science and Engineering, Maastricht University, Brightlands Chemelot Campus, Urmonderbaan 22, 6167 RD Geleen, The Netherlands
3 Laboratory Biomaterial, University of Applied Sciences Aachen, Heinrich-Mußmann-Straße 1, 52428 Jülich, Germany
4 Institute of Heat and Mass Transfer (WSA), RWTH Aachen University, Augustinerbach 6, 52056 Aachen, Germany
5 Department of Pneumology and Internal Intensive Care Medicine, Medical Clinic V, University Hospital RWTH Aachen, Pauwelsstraße 30, 52074 Aachen, Germany
* Correspondence: thiebes@ame.rwth-aachen.de (A.L.T.); jockenhoevel@ame.rwth-aachen.de (S.J.); Tel.: +49-241-80-47472 (A.L.T.); +49-241-80-47478 (S.J.)
† These authors contributed equally to this work.

Table S1. Percentage of positive ASCs (n=3) cells stained for eight mesenchymal stromal cell-characteristic marker of non-sprayed and sprayed with either gas-to-liquid ratio 2.5% or gas-to-liquid ratio 10% after 7 days of culture. Flow cytometric analysis shows that all experimental conditions fulfill the minimal criterion of Dominici et al. [16] regarding the surface marker expression.

	Percentage of Positive Cells Mean ± SD (%)					
	**Control**		**gas-to-liquid ratio 2.5%**		**gas-to-liquid ratio 10%**	
Minimal Criterion is ≥95%						
**CD90**	99.57	±0.29	99.63	±0.23	99.70	±0.17
**CD73**	99.90	±0.00	98.47	±2.48	99.90	±0.00
**CD105**	99.97	±0.06	99.97	±0.06	99.97	±0.06
Minimal Criterion is ≤2%						
**CD45**	0.00	±0.00	0.00	±0.00	0.00	±0.00
**CD34**	0.02	±0.03	0.03	±0.05	0.04	±0.06
**CD11b**	0.00	±0.00	0.00	±0.00	0.00	±0.00
**CD79α**	0.09	±0.01	0.09	±0.04	0.08	±0.02
**HLA-DR**	0.01	±0.00	0.01	±0.01	0.03	±0.02

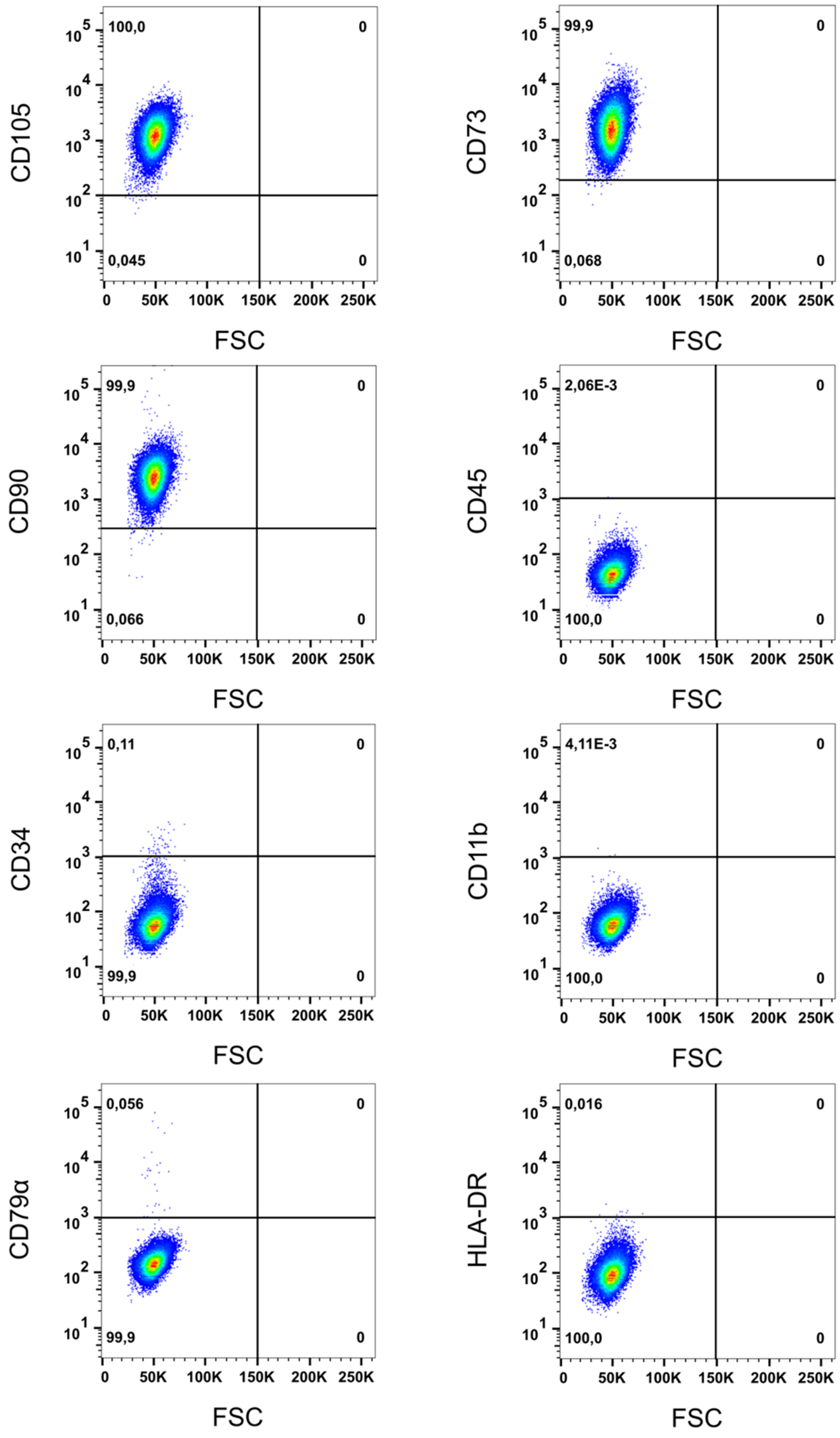

Figure S1. Dot plots of flow cytometry measurement (gas-to-liquid ratio 10%) for all examined cell surface markers. The x-axis of the dot plot shows the forward scatter value and the y-axis the intensity of the respective cell surface marker.

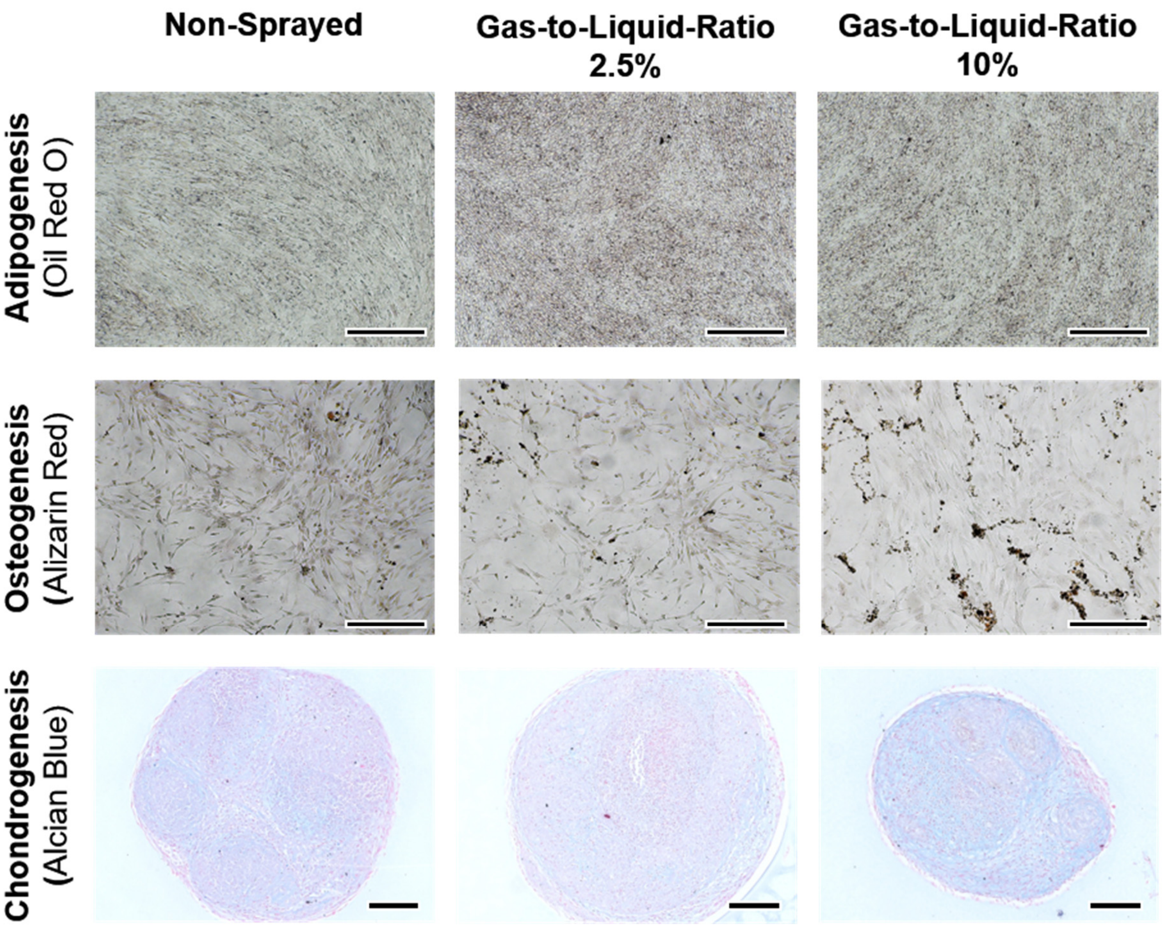

Figure S2. Non-sprayed and sprayed (gas-to-liquid ratio 2.5% and 10%) ASCs after culture with full growth medium and staining for adipocytes (top row), osteocytes (middle row, scale bars: 500 µm) and chondrocytes (bottom row, scale bar: 200 µm).
